# Fibroblast Heterogeneity in Inflammatory Bowel Disease

**DOI:** 10.3390/ijms252313008

**Published:** 2024-12-03

**Authors:** Bo-Jun Ke, Gabriele Dragoni, Gianluca Matteoli

**Affiliations:** 1 Translational Research Center for Gastrointestinal Disorders (TARGID), Department of Chronic Diseases and Metabolism, KU Leuven, 3000 Leuven, Belgium; bojun.ke@kuleuven.be (B.-J.K.); gabriele.dragoni@unifi.it (G.D.); 2 Gastroenterology Research Unit, Department of Experimental and Clinical Biomedical Sciences, University of Florence, 50134 Florence, Italy

**Keywords:** fibroblast heterogeneity, inflammatory bowel disease, fibroblast–immune interaction

## Abstract

Intestinal fibroblasts are pivotal players in maintaining tissue homeostasis and orchestrating responses to injury and inflammation within the gastrointestinal (GI) tract. Fibroblasts contribute significantly to the pathogenesis of inflammatory bowel disease (IBD), including Crohn’s disease and ulcerative colitis (UC), by secreting pro-inflammatory cytokines, modulating immune cell activity, and promoting fibrosis. In addition, fibroblasts play crucial roles in tissue repair and regeneration following acute injury or chronic inflammation. The dysregulation of fibroblast functions can lead to fibrotic complications, such as intestinal strictures and obstruction, which are common in advanced stages of IBD. Understanding the complex interplay between fibroblasts and other cell types in the intestine is essential to elucidate the underlying mechanisms of intestinal diseases and identify novel therapeutic targets. Future research aimed at deciphering the heterogeneity of intestinal fibroblasts and their dynamic roles in disease progression holds promise for the development of precision therapies to mitigate fibrosis and inflammation in intestinal disorders.

## 1. Introduction

Intestinal fibroblasts have crucial roles in maintaining tissue homeostasis and in participating in immune responses and tissue remodelling. However, in the context of response to injury and chronic insults, their aberrant activation contributes significantly to the pathogenesis of inflammatory bowel disease (IBD), both in Crohn’s disease and ulcerative colitis (UC). In the field of intestinal fibrosis, their dysregulation is associated with the occurrence of IBD complications, such as intestinal strictures and obstruction, which represent one of the major causes of treatment failures, particularly in Crohn’s disease. For this reason, recent research has sought to define the potential functions of these cells, such as secretion of pro-inflammatory cytokines, modulation of immune cell activity, and promotion of fibrosis, to tackle the consequences of pathogenic fibroblast activation. This heterogeneous differentiation in several mature subtypes complicates our understanding of fibroblast biology but may also represent an opportunity to selectively block specific activities while leaving others unchanged.

The focus of this review is to provide an overview of the current knowledge regarding intestinal fibroblast development and differentiation, including the different functions and interaction with immune cells of the several fibroblast subtypes that have been described. Additionally, we will discuss the present landscape of anti-fibrotic treatments and propose fibroblast targets to be considered for future clinical trials in light of the recent literature on fibroblast heterogeneity.

## 2. Heterogeneity of Fibroblasts in Development and Homeostasis

Mesenchymal stem cells (MSCs), originating from the mesodermal germ layer, are recognised for their multifaceted roles in maintaining tissue integrity [1]. These stromal cells offer structural support to organs, organising the synthesis and dynamic remodelling of the extracellular matrix (ECM). Additionally, they finely regulate processes involving tissue development, homeostasis, repair, and fibrosis. MSCs are multipotent stromal cells that can differentiate into a variety of cell types, including fibroblasts, osteoblasts, myocytes, adipocytes, and chondrocytes [2,3,4,5]. According to the guidelines of the International Society for Cellular Therapy, MSCs are characterised by the expression of CD105, CD73, and CD90, while they typically lack the expression of CD45, CD34, CD14 or CD11b, CD79α, CD19, and HLA-DR [6]. More recently, an increasing amount of evidence indicates the significant role played by platelet-derived growth factor receptor α (PDGFRA)-positive cells in mesoderm formation and the development of connective tissues during embryogenesis, firmly establishing PDGFRA as a key marker for MSCs [7,8].

In the small intestine of both humans and mice, a distinct population of Gremlin 1 (Grem1)^+^ cells has been recognised as intestinal MSCs. Through lineage tracing experiments conducted in Grem1-CreERT;R26-tdTomato mice, it has been observed that Grem1^+^ MSCs are located at the junction between the crypt and the villus, and ultimately contribute to the entirety of the small intestinal fibroblast compartment [9]. These self-renewing intestinal MSCs undergo differentiation into three distinct subsets of PDGFRA^+^ MSCs, a process regulated by BMP signalling [10]. More in detail, FOXL1^+^PDGFRA^high^ MSCs exhibit a primarily notable abundance at the base of villi, serving as a reservoir for BMP and CD81^+^PDGFRA^low^ MSCs that are beneath the crypts [11]. Remarkably, the latter secrete Grem1, an antagonist of BMP signalling, significantly impacting the physiology of the crypt-villus epithelium [12]. However, CD81^-^PDGFRA^low^ MSCs secrete Wnt antagonists and modulate epithelial stem cells via R-Spondin 1 (RSPO1) and RSPO3 [13,14].

MSCs are non-migratory cells, lack stress fibre formation, and have minimal force generation capability and a limited capacity to produce ECM proteins. Nonetheless, MSCs exhibit remarkable potential as progenitors of fibrogenic cells, with the high-capacity potential to differentiate into fibroblasts, which represent terminal cell types within the fibrogenic lineage [15]. Notably, connective tissue growth factor (CTGF) has been reported to be one of the key players for this differentiation from human bone marrow MSCs [16].

During human embryonic development in the forming villi, MSCs at the base of the villus undergo differentiation into WNT4^+^PDGFRA^low^ fibroblasts, thereby supporting epithelial formation [11]. Additionally, in the early stage of intestinal formation, C7^+^ASPN^+^ fibroblasts as fibrogenic progenitors differentiate into F3^high^DLL1^high^PDGFRA^high^ fibroblasts located at the bottom of villus and sub-epithelium [17]. These fibroblasts produce neuregulin 1 (NRG1), which increases the cellular diversity and maintains crypt-villus architecture [18]. At the same time of intestinal development, fibrogenic progenitors differentiate into ADAMDEC1^+^FABP^+^ fibroblasts in the submucosa [17]. These fibroblasts also produce several BMPs to promote stem cell maintenance [19]. In the later stage of development, GPX3^high^F3^low^PDGFRA^low^ fibroblasts appear in the villus core. However, the function of these fibroblasts has not been investigated yet [18].

## 3. Heterogeneity of Fibroblasts in Chronic Inflammation and Intestinal Fibrosis

Within the developed human intestine, fibroblasts located at the bottom of villi persist in maintaining epithelial stem cell function through WNT signalling. However, these fibroblasts are also associated with IBD by disrupting the integrity of the epithelial barrier [20,21]. In contrast to the development phase, ADAMDEC1^+^ fibroblasts in the adult intestine are predominantly located in the lamina propria and have been identified as pro-inflammatory subset in UC due to their IL-11 production [20,22]. These fibroblasts also play a crucial role in preventing the abnormal accumulation and disorganisation of ECM proteins [23]. Another important subset of fibroblasts, CD34^+^CD90^+^GREM1^+^ fibroblasts, positioned at the crypt-bottom and submucosa, rapidly respond to intestinal injury by up-regulating niche factors that maintain a specific environment for intestinal epithelial stem cells, such as GREM1, RSPO1, CCL2, and amphiregulin (AREG) [24]. Increased levels of GREM1 have been observed in the fibro-stenotic ileum of Crohn’s disease patients [25]. This alteration could stimulate vascular endothelial growth factor receptor 2 (VEGFR2), leading to downstream MAPK signalling and promoting fibroblast proliferation and activation [26]. PDGFRA is a marker associated with pan fibroblasts. Notably, elevated levels of PDGFRA in fibroblasts are connected to their proliferation and transition into myofibroblasts [27]. Moreover, PDGFRA^low^ crypt-bottom fibroblasts facilitate the proliferation of intestinal stem cells, whereas PDGFRA^high^ crypt-bottom fibroblasts drive the differentiation of epithelial cells to maintain tissue integrity [28].

Fibroblast activation protein (FAP)^+^ TWIST1^+^ fibroblasts are integral to the pathogenesis of fibro-stenotic Crohn’s disease. Two recent studies have identified FAP^+^ fibroblasts as the predominant subtype responsible for the extensive production of ECM within the Crohn’s-affected ileum [29,30]. These studies have elucidated that the transcription factor TWIST1 plays a crucial role in the activation of FAP^+^ fibroblasts following pro-fibrotic signalling. This activation is modulated by CD150^+^ monocytes and CXCL9^+^ macrophages. Furthermore, signalling pathways involving tumour necrosis factor alpha (TNFα), the interleukin 1 (IL-1) family, transforming growth factor-beta (TGF-β), oncostatin M (OSM), and interferon-gamma (IFN-γ) have been implicated in the regulation of fibroblast activation. Importantly, FAP^+^ fibroblasts have also been suggested as a key subset defining a group of IBD patients who do not respond to anti-TNFα. This process is driven by IL-1 signalling, which is modulated by neutrophils [31]. Although CD34^+^GREM1^-^, CD34^+^GREM1^+^, and FGFR2 fibroblasts have been proposed as potential precursors to FAP+ fibroblasts, the exact origin of these FAP^+^ fibroblasts has not yet been validated.

Alpha-smooth muscle actin (α-SMA)^+^ myofibroblasts possess robust capabilities in collagen production and contraction, driving the fibrotic process through distinct mechanisms. Primarily, these activated α-SMA^+^ myofibroblasts secrete ECM proteins, along with various cytokines and chemokines, directly and indirectly contributing to the thickening of the bowel wall [32]. In Crohn’s disease patients, primary fibroblasts isolated from stenotic ileum exhibit increased ECM organisation and collagen production when compared to myofibroblasts from normal and inflamed areas. Notably, stenotic myofibroblasts were identified to enhance tissue stiffness while concurrently suppressing matrix metalloproteinase 3 (MMP-3) activity expression, thereby promoting the development of fibrosis [33]. The transition of pericytes to myofibroblasts has been proposed as a contributing factor in intestinal fibrosis. This differentiation is driven by a complex network of signalling pathways, with TGF-β serving as a potent inducer of myofibroblast differentiation and platelet-derived growth factor (PDGF), promoting pericyte proliferation and migration [34]. Through these pathways, pericytes adopt a myofibroblast phenotype, characterized by increased expression of α-SMA and enhanced ECM production.

Smooth muscle cells (SMCs) and fibroblasts arise from the same mesenchymal cell lineage, with SMCs considered progenitors of myofibroblasts [35]. In response to various stimuli, SMCs exhibit plasticity in altering their phenotypes, as demonstrated by the morphological and contractile activity changes in SMCs isolated from the ileum of Crohn’s disease patients. These Crohn’s-derived SMCs displayed an overexpression of PDGF-β, leading to a transition from a myogenic to a synthetic phenotype [36]. This shift occurred simultaneously with a reduction in contractile response to maintain a quiescent smooth muscle state. Furthermore, SMCs from Crohn’s ileum release substantial amounts of IL-6, contributing to inflammatory processes. Additionally, these cells actively contribute to the development of intestinal fibrosis by inducing the production of collagens and MMPs [37].

In addition to SMCs, adipocytes also originate from the mesenchymal cell lineage. Preadipocyte factor 1, also known as Dlk1/FA1, is expressed in pre-adipocytes and has been found as a marker in cells that have the capability to transdifferentiate into myofibroblast-like cells. These cells express α-SMA and various collagen proteins, contributing to the fibrotic process. Furthermore, in a related study, it has been suggested that bacterial-derived lipopolysaccharide (LPS) signalling through Toll-like receptor 4 (TLR4) and TGF-β signalling are proposed as key pathogenic pathways in this context [38]. Similar mechanisms have been observed in the fibrotic environment of creeping fat, highlighting their significance in the pathogenesis of intestinal fibrosis [39].

Epithelial–mesenchymal transition (EMT) and endothelial–mesenchymal transition (EndoMT) have also been indicated in the pathogenesis of intestinal fibrosis. These complex cellular processes involve the phenotypic transformation of epithelial cells or endothelial cells, leading to the acquisition of mesenchymal characteristics, including increased migratory and invasive capabilities. Within the context of intestinal fibrosis, EMT and EndoMT play a role in generating activated fibroblasts, crucial contributors to the deposition of ECM proteins. The induction of EMT and EndoMT is regulated by various signalling pathways, such as TGF-β, Wnt, and Notch, in response to inflammatory stimuli or tissue injury. Nevertheless, there is currently limited concrete evidence supporting the occurrence of EMT and EndoMT in the human intestine.

**Figure 1:**
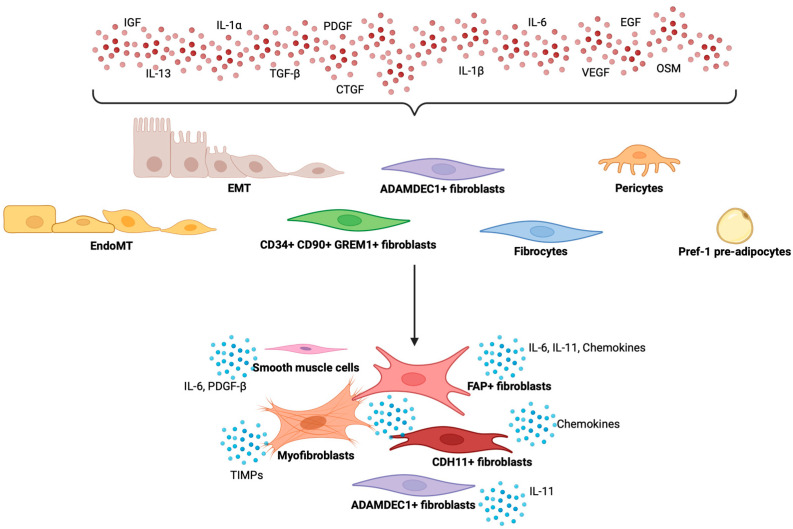
Potential sources of pathogenic fibroblasts and pro-fibrotic signalling pathways in Crohn’s disease are multifaceted and stem from a complex interplay of factors. These fibroblasts can originate from various cellular populations, including resident fibroblasts, fibrocytes, and even EMT or EndoMT processes within the intestinal epithelium. In the context of Crohn’s disease, inflammatory cytokines such as TNFα and IL-1β play a crucial role in activating these fibroblasts, promoting their proliferation and the secretion of ECM components. Additionally, signalling pathways, including TGF-β and Wnt/β-catenin, are implicated in the transition of these fibroblasts into myofibroblasts, which further contribute to tissue fibrosis and the characteristic scarring observed in affected individuals. Activated fibroblasts can also exacerbate inflammation and fibrosis by releasing various chemokines and cytokines, which attract additional immune and non-immune cells to the site.

Despite these insights, a comprehensive understanding of fibroblast heterogeneity and their involvement in the pathogenesis of the deeper layers of the bowel wall remains elusive (Figure 1). However, selective modulation of one or more of these fibroblast subtypes may be tested with the aim of reducing the fibrotic burden associated to chronic insult and inflammation.

## 4. Immune-Stromal Cell Interaction in Intestinal Fibrosis

The dynamic interplay between innate immunity and fibroblasts plays a crucial role in shaping both inflammatory and fibrotic responses. Upon exposure to microbial stimuli or tissue damage, innate immune cells, such as macrophages and dendritic cells, set off an inflammatory cascade. This, in turn, prompts the release of various key mediators in the activation and differentiation of fibroblasts, including TGF-β, TNFα, and interleukins. A more detailed perspective reveals that neutrophil-dependent IL-1 signalling may be instrumental in mediating the activation of IL-1R+ fibroblasts. Notably, these IL-1-driven interactions between fibroblasts and neutrophils have been implicated in the context of resistance to biological treatments [31]. Of note, recent evidence also suggests neutrophil extracellular traps as potential early drivers of fibrogenesis by direct activation of intestinal fibroblasts derived from Crohn’s disease patients [40].

Additionally, the release of OSM by myeloid cells can initiate fibrotic processes by activating JAK/STAT, MAPK, and PI3K signalling pathways in fibroblasts [41]. In response, activated fibroblasts release several chemokines and chemoattractants (CCL2 and CCL7), creating a positive feedback loop that recruits more myeloid cells to the site of wound healing or fibrotic lesions [42]. Recent studies using scRNA-seq have identified the potential involvement of CD209^+^CD68^+^ macrophages, neuregulin 1 (*NRG1*)^+^ macrophages, and inflammatory fibroblasts in the pathophysiology of the disease. Additionally, the presence of angiogenic neutrophils has been demonstrated in inflamed human tissue in the same study. However, a comprehensive understanding of the origin and function of these neutrophils in the gut remains elusive [43]. An elevated quantity of CD16^+^CD206^+^ macrophages has been identified in the mucosa affected by Crohn’s disease, in contrast to healthy colon. This specific macrophage subset has the potential to increase susceptibility to intestinal fibrosis through STAT6 activity and involvement in Wnt signalling [44].

Adaptive immunity, particularly T cells, also plays a role in promoting fibroblast activation. While Th1 immunity is traditionally considered anti-fibrotic, Th1 cells produce pro-inflammatory cytokines such as IFNγ and TNFα. These cytokines have been demonstrated to induce fibroblast activation, modulate ECM through MMPs, and enhance TGF-β1 production in activated fibroblasts, thereby intensifying fibrogenesis. In contrast, Th2 and Th17 immunity are recognised as pro-fibrotic subsets. Th2 immunity produces IL-4, IL-5, IL-13, and IL-33, which are crucial for TGF-β signalling but do not influence other pro-fibrotic factors like IL-6, IL-8, α-SMA, and collagen I. Th17 immunity relies on interleukins and TGF-βs, produced by other immune cells and fibroblasts. The derived cytokine IL-17 is essential for fibroblast activation and collagen production in fibroblasts through the STAT3 signalling pathway. In response, activated fibroblasts release chemokines, chemoattractants, and various cytokines to recruit additional T cells and influence their phenotype 117 [31,39].

## 5. Strategies for Targeting Fibroblast Activity

To date, no dedicated medication has been developed for the specific targeting of intestinal fibrosis. Nevertheless, there are two FDA-approved anti-fibrotic drugs, nintedanib and pirfenidone, which have found application in the treatment of progressive idiopathic pulmonary fibrosis (IPF). Nintedanib is a growth factor pathway-targeting inhibitor, specifically targeting RTKs such as FGFRs, VEGFRs, and PDGFRs [45]. In in vitro study using LPS-induced Caco-2 cells, nintedanib demonstrated a significant inhibitory effect on both mRNA and protein levels of CEBPB, PCK1, and EFNA1. Furthermore, in the same study, nintedanib substantially ameliorated DSS-induced acute colitis in mice by impeding the CEBPB/PCK1 and CEBPB/EFNA1 signalling pathways [46]. In the context of intestinal stenosis, a key characteristic is the hyperplasia of intestinal smooth muscle, which is believed to contribute to bowel obstruction. Notably, in mice with TNBS-induced colitis, nintedanib exhibited the ability to reduce the hyperplasia of intestinal SMCs and to inhibit the expression of the pro-inflammatory cytokines TNFα and IL-1β [47].

Pirfenidone, another FDA-approved anti-fibrotic drug, acts by inhibiting both the synthesis and activation of TGF-βs [48]. Like nintedanib, pirfenidone has shown to exhibit anti-fibrogenic properties in a murine colitis model induced by DSS [49]. Pirfenidone effectively reduced the deposition of collagen in colitis-associated fibrosis and significantly suppressed the mRNA expression of *COL1A2*, *COL3A1*, and *TGFB1*. Furthermore, pirfenidone inhibited the activation of TGF-β-related Smad and MAPK pathways, signifying its potential as an anti-fibrotic agent in the context of intestinal fibrosis in mice [49]. In a radiation-induced intestinal fibrosis rat model, pirfenidone showed significantly attenuated fibrotic lesions in irradiated intestines. This effect was attributed to the inhibition of the TGF-β1/Smad/CTGF pathway, resulting in reduced collagen deposition and promising outcomes in the context of intestinal fibrosis [50]. While murine models have shown the potential anti-fibrotic benefits of nintedanib and pirfenidone in the intestine, it is noteworthy that both these agents have reported gastrointestinal adverse effects when used in IPF patients, such as diarrhoea and pancolitis [51,52].

In addition to nintedanib and pirfenidone, numerous other anti-fibrotic medications are currently undergoing clinical trials at various phases, each tailored to target a spectrum of fibrotic disorders. Notably, a phase 3 clinical trial (NCT03955146) is presently underway to assess the effectiveness and safety of pamrevlumab, a recombinant antibody-targeting CTGF, in individuals with IPF. Encouragingly, in a mouse model of lung fibrosis induced by thoracic irradiation, pamrevlumab demonstrated superior monotherapy in inhibiting progressive lung remodelling and preserving lung function compared to nintedanib or pirfenidone after 8 weeks of treatment [53]. In a recent study, a notable increase in *CTGF* mRNA expression was observed in resected small intestine samples obtained from patients with Crohn’s disease. Particularly, *CTGF* mRNA was found to be localised predominantly in fibroblasts situated within the submucosal layer, in proximity to lymph follicles, and in regions displaying severe damage near the luminal surface [54]. A phase 2 clinical trial (NCT05843578) is presently ongoing to assess the safety, pharmacokinetics, and pharmacodynamics of AGMB-129 in patients with fibro-stenotic Crohn’s disease. This GI-restricted inhibitor of TGFβR1 (ALK5) is specifically engineered to target TGFβ signalling, an essential regulator of fibrosis, with the goal of decreasing the necessity for bowel resection surgery in individuals with fibro-stenotic Crohn’s disease [55]. Tackling TNF-like cytokine 1A (TL1A) activity also holds great expectations, as the TL1A axis has shown to be involved in both intestinal inflammation and fibrosis [56]. Several phase 2 trials and one phase 3 clinical trial are underway to address the anti-inflammatory implications of blocking this pathway, leading the way to test its anti-fibrotic potential in the near future [56].

Various FGF analogues are currently undergoing investigation, with pegbelfermin, also identified as BMS-986036, displaying promising outcomes in a clinical trial. Notably, at the 24-week mark, this PEGylated human FGF21 analogue demonstrated improvements in serum fibrogenesis biomarkers in patients with non-alcoholic steatohepatitis [57,58]. In a phase 2 trial, aldafermin, an FGF19 analogue, effectively reduced lipid deposition in the liver and exhibited a trend toward improving fibrosis [59]. The potential advantages of pegbelfermin and aldafermin may be particularly relevant for individuals with fibro-stenotic Crohn’s disease, characterised by elevated levels of FGF and VEGF in their serum during active disease states. On the other hand, several promising pre-clinical targets have then failed to show benefits in fibro-stenotic Crohn’s disease trials, such as the IL-36R inhibitor spesolimab (NCT05013385).

Interestingly, various targets used for IBD have been explored for their potential anti-fibrotic effects in other organs. One such example is ustekinumab, a human monoclonal antibody targeting IL-12 and IL-23, which has been used in the treatment of moderate to severe IBD since 2016 [60]. A proof-of-concept study assessed the anti-fibrotic effects of ustekinumab in primary biliary cirrhosis, revealing no significant impact on fibrosis in this context [61]. Another example is RXC008, a gastrointestinal-targeted ROCK inhibitor developed as a potential first-in-class treatment for fibro-stenotic Crohn’s disease. In a pre-clinical study, RXC008 demonstrated anti-fibrotic effects in adoptive T-cell transfer and chronic DSS murine models of colitis, suppressing fibrosis and reducing the expression of fibrotic markers such as *COL1A1*, *COL1A2*, and *TGFB1*. Furthermore, another study using RXC008 demonstrated a reduction of intestinal fibrosis by diminishing MRTF and p38 MAPK activation and simultaneously increasing autophagy in intestinal fibroblasts [62]. Considering the extensive and solid pre-clinical evidence, a Phase 1 clinical study with RXC008 has just started, with first results expected by the end of 2024. Additionally, the selective ROCK2 inhibitor, RXC007 (currently in clinical trial NCT05570058), has exhibited anti-fibrotic efficacy in lung tissues, showcasing its potential across multiple organ systems, as evidenced by reductions in the Ashcroft score, trichrome, picrosirius red staining, and downregulation of fibrotic genes including *CTGF* and *FN1* [63].

In the past decade, there has been a growing focus on JAK inhibitors as a novel therapeutic avenue in IBD. Preclinical models have indicated that tofacitinib, a pan-JAK inhibitor, can mitigate liver fibrosis in mice with autoimmune hepatitis and bleomycin-induced skin fibrosis [64]. Although tofacitinib has demonstrated efficacy in UC, its effects on Crohn’s disease were found to be disappointing, as the phase 2a trial (NCT00615199) revealed no significant differences in clinical response or remission between the placebo group and the treatment groups [65]. Filgotinib, a JAK1/JAK2 inhibitor, has been administered to patients with rheumatoid arthritis (RA) since 2016. Given the similarities between RA and Crohn’s disease, the efficacy of filgotinib in Crohn’s disease was assessed. Unfortunately, it did not progress beyond phase III clinical trials (NCT02914561) [66,67]. Additionally, another JAK1/JAK2 inhibitor, ruxolitinib, has received approval for use in patients with intermediate- and high-risk myelofibrosis, a condition characterised by overactive bone marrow leading to the development of scar tissue [68,69]. More recently, both the FDA and EMA have granted approval for upadacitinib, a JAK1-selective inhibitor, for use in patients with Crohn’s disease [70]. Collectively, the diverse range of JAK inhibitors holds promise as a novel avenue for exploring anti-fibrotic therapeutic options.

The complex progression of fibrosis requires the involvement of diverse cells and signalling pathways. Ongoing developments in drugs targeting these aberrant pathways show promising anti-fibrotic properties in clinical trials (Table 1). Nonetheless, the frequent occurrence of side effects often necessitates the discontinuation of these drugs, presenting a significant challenge in drug development. Mitigating adverse effects remains a crucial aspect of enhancing the efficacy of anti-fibrotic medications. Moreover, given the complex interplay of signalling pathways in fibrosis, the exploration of multitarget drug regimens emerges as a beneficial strategy for more effective fibrosis therapy. In the intestine, a shared definition of fibrosis improvement is necessary in the context of clinical trials to develop effective medications with clear anti-fibrotic properties. The next challenge would then be to understand the ideal positioning of anti-fibrotic treatments in the natural history of Crohn’s disease.

## 6. Conclusions

Understanding fibroblast heterogeneity and the complex mechanisms that establish intestinal fibrosis is an open challenge. As fibroblasts represent the key cell type involved in the process of fibrogenesis, a direct blocking of one (or more of) pathological subtype would be regarded as one of the best options in the context of anti-fibrotic targets. As no clinical studies in this direction are ongoing in fibro-stenotic Crohn’s disease, future clinical trials should move towards this direction, aiming at extremely selective anti-fibrotic drugs.

## Figures and Tables

**Table 1:** Summary of anti-fibrotic medication.

Medication	Target	Status	Tissue	Outcomes	Main Side Effects
Nintedanib [71]	RTKs	Approved	IPF	Reduction of FVC decline	Diarrhoea, nausea
Pirfenidone [72,73]	TGF-βs	Approved	IPF	Reduction of FVC decline	Nausea, rash, headache, dyspepsia
Pamrevlumab [74]	CTGF	Phase III ongoing (NCT03955146)	IPF	Reduction of FVC decline *	Fatigue, urinary tract infections *
Ruxolitinib [75]	JAK1/JAK2	Approved	Myelofibrosis	Reduction of spleen size and debilitating symptoms, Increase of OS	Anaemia, thrombocytopenia
AGMB-129	TGFβR1	Phase II ongoing (NCT05843578)	Fibro-stenotic CD	Unknown	Unknown
Pegbelfermin [57]	FGF21	Phase II (NCT03486899)	Liver fibrosis	Primary endpoint not met	Unknown
Aldafermin [76]	FGF19	Phase II (NCT03912532)	Liver fibrosis	Improvement of liver stiffness and aminotransferases	Diarrhoea
RXC008	ROCK	Phase II ongoing (RD783.35406)	Fibro-stenotic CD	Unknown	Unknown
RXC007	ROCK2	Phase II ongoing (NCT05570058)	IPF	Unknown	Unknown
TEV-48574 [56]	TL1A	Phase II (NCT05499130)	IBD	Unknown	Unknown

CD: Crohn’s disease; FVC: forced vital capacity; IBD: inflammatory bowel disease; IPF: idiopathic pulmonary fibrosis; OS: overall survival. * Phase II data, Phase III ongoing.
